# HIV-1 capsid stability and reverse transcription are finely balanced to minimize sensing of reverse transcription products *via* the cGAS-STING pathway

**DOI:** 10.1128/mbio.00348-24

**Published:** 2024-03-26

**Authors:** Jenna E. Eschbach, Maritza Puray-Chavez, Shawn Mohammed, Qiankun Wang, Ming Xia, Lin-Chen Huang, Liang Shan, Sebla B. Kutluay

**Affiliations:** 1 Department of Molecular Microbiology, Washington University in St. Louis, St. Louis, Missouri, USA; 2 Division of Infectious Diseases, Department of Medicine, Washington University School of Medicine, Saint Louis, Missouri, USA; 3 Andrew M. and Jane M. Bursky Center for Human Immunology and Immunotherapy Programs, Washington University School of Medicine, Saint Louis, Missouri, USA; Dana-Farber Cancer Institute, Boston, Massachusetts, USA

**Keywords:** HIV-1, capsid, innate immunity, innate sensing, capsid stability, reverse transcription, lenacapavir, cGAS

## Abstract

A critical determinant for early post-entry events, the HIV-1 capsid (CA) protein forms the conical core when it rearranges around the dimeric RNA genome and associated viral proteins. Although mutations in CA have been reported to alter innate immune sensing of HIV-1, a direct link between core stability and sensing of HIV-1 nucleic acids has not been established. Herein, we assessed how manipulating the stability of the CA lattice through chemical and genetic approaches affects innate immune recognition of HIV-1. We found that destabilization of the CA lattice resulted in potent sensing of reverse transcription products when destabilization *per se* does not completely block reverse transcription. Surprisingly, due to the combined effects of enhanced reverse transcription and defects in nuclear entry, two separate CA mutants that form hyperstable cores induced innate immune sensing more potently than destabilizing CA mutations. At low concentrations that allowed the accumulation of reverse transcription products, CA-targeting compounds GS-CA1 and lenacapavir measurably impacted CA lattice stability in cells and modestly enhanced innate immune sensing of HIV. Interestingly, innate immune activation observed with viruses containing unstable cores was abolished by low doses of lenacapavir. Innate immune activation observed with both hyperstable and unstable CA mutants was dependent on the cGAS-STING DNA-sensing pathway and reverse transcription. Overall, our findings demonstrate that CA lattice stability and reverse transcription are finely balanced to support reverse transcription and minimize cGAS-STING-mediated sensing of the resulting viral DNA.

**IMPORTANCE:**

In HIV-1 particles, the dimeric RNA genome and associated viral proteins and enzymes are encased in a proteinaceous lattice composed of the viral capsid protein. Herein, we assessed how altering the stability of this capsid lattice through orthogonal genetic and chemical approaches impacts the induction of innate immune responses. Specifically, we found that decreasing capsid lattice stability results in more potent sensing of viral reverse transcription products, but not the genomic RNA, in a cGAS-STING-dependent manner. The recently developed capsid inhibitors lenacapavir and GS-CA1 enhanced the innate immune sensing of HIV-1. Unexpectedly, due to increased levels of reverse transcription and cytosolic accumulation of the resulting viral cDNA, capsid mutants with hyperstable cores also resulted in the potent induction of type I interferon-mediated innate immunity. Our findings suggest that HIV-1 capsid lattice stability and reverse transcription are finely balanced to minimize exposure of reverse transcription products in the cytosol of host cells.

## INTRODUCTION

In mature HIV-1 particles, the viral core is formed when the cleaved capsid (CA) domain of the major structural protein Gag forms a conical lattice (herein referred to as the CA lattice) around the condensed viral RNA genome and associated viral proteins and enzymes (1–3). The mature HIV-1 core contains hexameric and pentameric rings of CA that are stabilized through an extensive network of intra- and inter-subunit interactions (4–9) and small molecules such as inositol hexakisphosphate (IP_6_) (10, 11). In target cells, reverse transcription initiates within this lattice, which ultimately uncoats by partial shedding of CA to allow integration of the viral cDNA into the host cell chromosome (12–15). Both biochemical and imaging studies indicate that a comparably small fraction of virion-associated CA remains associated with reverse transcription complexes, which play a critical role in subsequent steps in HIV-1 replication including reverse transcription and nuclear entry (12–15). The timing, extent, and subcellular location of uncoating are not only critically important for the early steps of HIV-1 replication but also can generate pathogen-associated molecular patterns (PAMPs) that trigger innate immune activation (12–17).

A number of cytosolic pattern recognition receptors (PRRs) have been demonstrated to contribute to the detection of HIV-1 replication intermediates including DNA sensors cyclic GMP-AMP synthase (cGAS) (18–21) and IFI16 (22, 23), and RNA sensors DDX3 (24) and MDA5 (25). In addition, the accessory proteins PQBP1 (26, 27) and NONO (28) can potentiate cGAS-mediated sensing of incoming virions, and intron-containing HIV-1 RNAs can be detected by currently unknown sensors in a MAVS-dependent manner during late stages of infection (29, 30). Involvement of these receptors in sensing of HIV-1 replication intermediates appears to be complex, cell type dependent, and largely limited to myeloid cells, which are less efficiently infected with HIV-1 compared to the activated CD4+ T cells that represent the main target of HIV-1 *in vivo*. By contrast, HIV-1 replication in CD4+ T cells does not appear to trigger innate immune activation despite the presence of an active cGAS-STING pathway (31). Nonetheless, recent studies suggest that efficient evasion of cGAS-mediated innate immune activation may underlie the spread of pandemic Group M HIV-1 (32), and naturally occurring mutations in CA can impact cGAS-sensitivity (33).

Several studies have revealed the critical role of CA in innate immune sensing of HIV-1 replication intermediates. For example, incoming wild-type (WT) HIV-1 does not appear to trigger significant innate immune induction in primary monocyte-derived macrophages (MDMs) (20, 34, 35), which is often ascribed to the protective role of the CA lattice. In the more commonly used THP-1 monocytic cell line model, WT HIV-1 has been reported to induce a range of innate immune responses, likely due to differences in cell culture and stimulation conditions, virus preparation, and the effective multiplicity of infection (MOI) (18, 33, 35–38). In THP-1 cells, CA mutations that prevent the recruitment of cellular cofactors CPSF6 and cyclophilin A (20) or alter CA-sp1 proteolytic cleavage (35) can enhance innate immune sensing, whereas IP6-mediated stabilization of the CA lattice (39) or CA mutations that confer resistance to the destabilizing effects of the CA-targeting compound PF74 can result in evasion of sensing (33). Unequivocally, cGAS-STING-mediated recognition of incoming viruses seems to be the main pathway that triggers innate immune activation in HIV-1-infected THP-1 cells and macrophages (18, 33, 35–39). This response can be further potentiated by the depletion of the cellular exonuclease TREX1, which degrades reverse transcription byproducts (20, 37, 40), and the CA-binding protein PQBP1, which bridges cGAS and the incoming viral cores (26, 27).

Until recently, it was widely believed that uncoating of the viral core initiates in the cytosol soon after entry and is accelerated by reverse transcription (41–48). However, recent imaging- and cryo-EM-based studies highlighted that in a small fraction of incoming virions, the conical CA lattice may remain associated with the reverse transcription complexes throughout the cytosol and directly mediate the nuclear entry of viral DNA by interacting with the nuclear pores (49–57). If so, it is predicted that cytosolic nucleic acid sensors may not access the viral PAMPs. In addition to its structural role, CA plays a key regulatory role in multiple steps of the viral life cycle (58–63). As a result, mutations or compounds that target the critical interactions between individual CA subunits disrupt particle assembly, virion morphogenesis, reverse transcription, and nuclear entry in target cells (15, 59, 64–68), which may also impact innate immune sensing.

It is thus intuitive that destabilization of the CA lattice can result in differential innate sensing of the prematurely exposed viral RNAs and/or unprotected reverse transcription intermediates. Although modifications in CA have been linked to altered innate immune activation, a clear link between CA lattice stability and innate immune sensing has not been established. We have previously shown that destabilization of the CA lattice results in premature exposure and degradation of the viral genome in target cells, in part explaining the well-described reverse transcription defects of these viruses (69). Whether tampering with CA lattice stability results in sensing of the viral replication intermediates prior to their degradation remains unknown.

In this study, we assessed how manipulation of core stability chemically, through potent CA-targeting compounds, and genetically, through mutations that inhibit inter- and intra-hexamer interactions, alters innate immune sensing. We found that destabilization of the CA lattice results in more potent sensing reverse transcription products, but not the incoming viral genomic RNA, provided that destabilization itself does not hinder reverse transcription. Unexpectedly, due to the combined effects of enhanced reverse transcription and accumulation of these products in the cytosol, two separate hyperstable capsid mutants induced innate immune activation most potently. The highly potent CA-targeting compounds GS-CA1 and lenacapavir modestly enhanced innate immune sensing of WT HIV-1 at low concentrations, but eliminated it at concentrations that blocked reverse transcription. Notably, innate immune activation observed with viruses containing unstable cores was abolished by low concentrations of lenacapavir. Finally, we found that all CA mutants induced innate immune activation through the cGAS-STING-mediated sensing of reverse transcription products and without the involvement of RNA sensing pathways. Overall, our findings demonstrate that CA lattice stability is finely balanced to allow reverse transcription and minimize cGAS-STING-mediated sensing by providing physical protection and mediating efficient nuclear entry of the viral cDNA.

## RESULTS

### HIV-1 reverse transcription products induce innate immune activation in the THP-1 monocytic cell line

**Fig 1:**
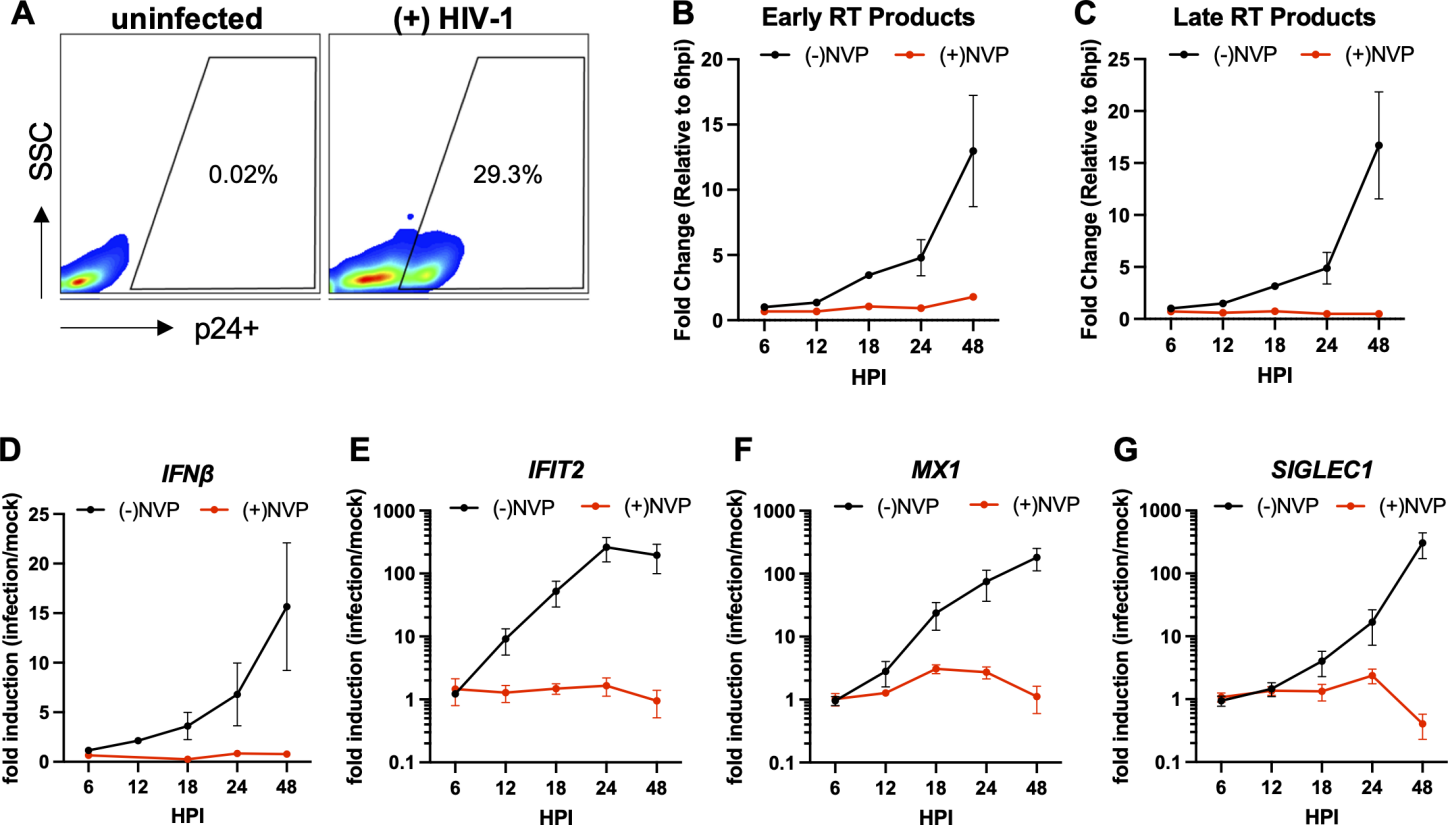
HIV-1 reverse transcription products induce innate immune activation. THP-1 cells were infected with HIV-1_NL4-3_/VSV-G at an MOI 5 i.u./cell (titered on TZM-bL cells) in the absence or presence of 25 µM nevirapine (NVP). (**A**) Infected cells were fixed at 48 hpi, stained for p24 (CA), and analyzed by flow cytometry as detailed in Materials and Methods. (**B–G**) Accumulation of early RT products (**B**), late RT products (**C**), and expression of IFN-β (**D**), and ISGs including *IFIT2*, *MX1,* and *SIGLEC1* (**E–G**) were analyzed by qPCR at the indicated time points post-infection. Levels of early and late RT products in panels B and C were normalized relative to 6 hpi/(-)NVP samples. The expression of IFN-β and ISG in D–-G was normalized relative to mock-infected cells. Data are derived from *n* = 2–7 independent biological replicates. Graphs show the mean with error bars displaying the SEM.

The THP-1 monocytic cell line is widely utilized to study innate sensing of HIV-1 (18, 26, 28, 33, 35–39) and represents a model for HIV-1 infection in myeloid cells. Since we and others found that stimulation of THP-1 cells with phorbol-12-myristate-13-acetate results in substantially lower rates of infection (18), we used unstimulated (and hence cycling) THP-1 cells in our studies. Inoculation of THP-1 cells with HIV-1/VSV-G at an MOI of 5 i.u./cell (titered on the TZM-bL indicator cell line) resulted in infection of ~25%–30% of the cells (Fig. 1A; Fig. S1A). Demonstrating the overall restrictive nature of this cell line model, accumulation of HIV-1 early and late reverse transcription products (herein RT products) was considerably slow, peaking at 24 hpi (Fig. 1B and C) and coinciding with the appearance of 2-LTR circles and integrated viral DNA (Fig. S1B and C). WT HIV-1 infection has been reported to induce low expression levels of type-I interferons (IFNs) and interferon-stimulated genes (ISGs) (33, 35, 36). Under our experimental conditions, we found induction of IFN-β (Fig. 1D) and several ISGs (i.e., *IFIT2*, *MX1*, *SIGLEC1, ISG15, CXCL10*) as early as 12–18 hpi, with levels peaking at 24 and 48 hpi, concurrent with the accumulation of reverse transcription intermediates (Fig. 1E through G; Fig. S1D through I). Despite the considerable variability in the magnitude of ISG induction (Fig. S1F through I), the observed innate immune activation completely depended on reverse transcription, as the NNRTI nevirapine (NVP) prevented the induction of IFN-β and ISGs (Fig. 1D through G; Fig. S1D through I). Differences in virus preparation methods have recently been shown to impact innate sensing of HIV-1 (38). Of note, the results presented herein have been derived from multiple virus preparations (using different plasmid stocks) and under previously described conditions that minimize lipopolysaccharide (LPS) contamination (38) (also see Materials and Methods section). The sensitivity of innate immune activation to nevirapine further argues against plasmid DNA and LPS contamination in virus preparations contributing to the observed response.

### Impact of CA-targeting compounds GS-CA1 and lenacapavir on innate immune sensing

**Fig 2:**
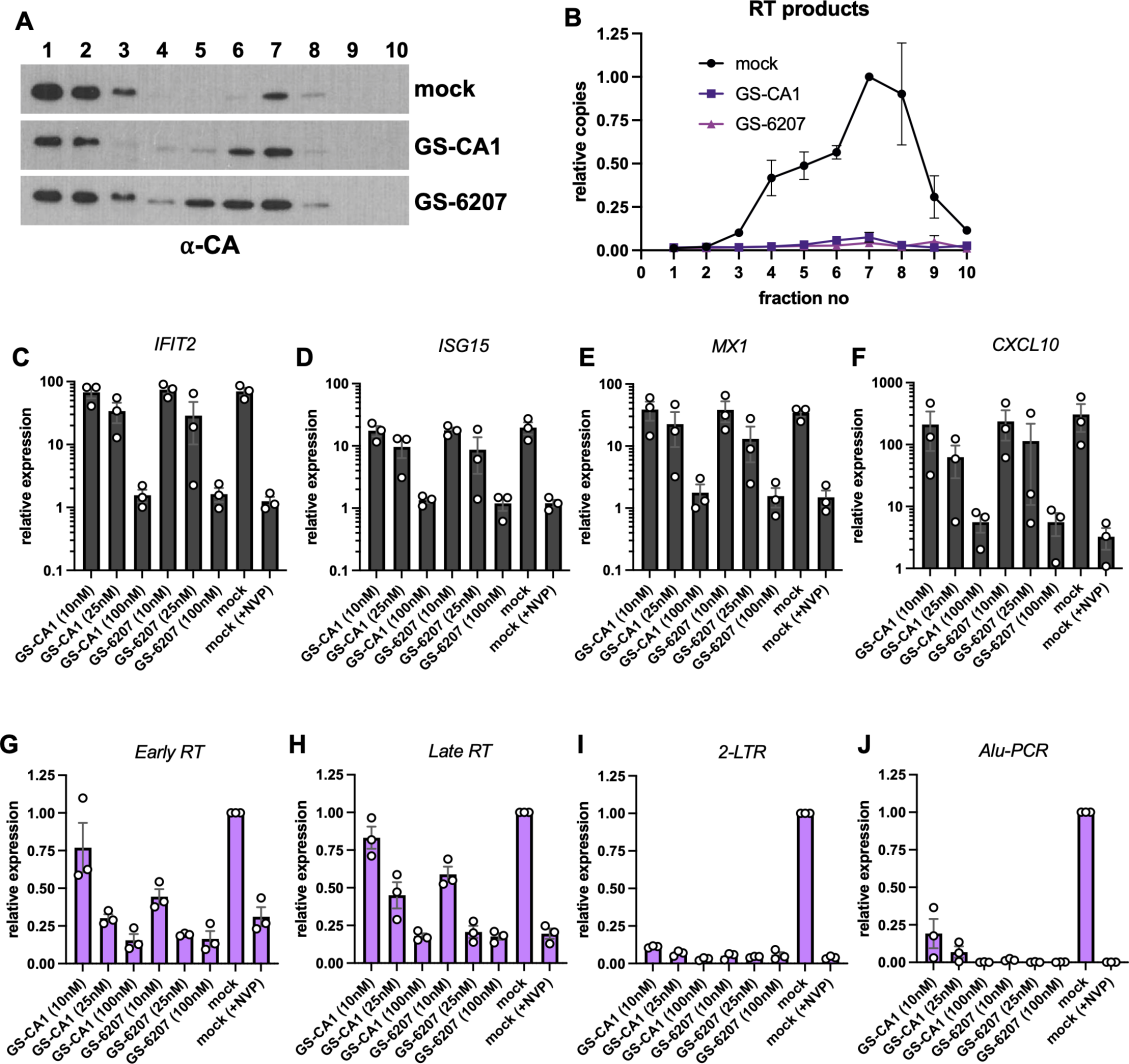
Lenacapavir (GS-6207) and GS-CA1 affect innate immune sensing of HIV-1. (**A, B**) PgsA-745 cells were synchronously infected with VSV-G-pseudotyped GFP reporter HIV-1 in the presence of 100  nM GS-CA1 or lenacapavir (GS-6207). Cells were processed at 2 hpi, as explained in Materials and Methods. Collected fractions were analyzed by western blotting using antibodies against CA-p24 (**A**) or subjected to qPCR for detection of viral reverse transcription products (**B**). Immunoblots in A are representative of three independent experiments. Data in B show the normalized copies of reverse transcription products relative to mock-treated samples, whereby the peak core fraction, fraction 7, is set to 1. Data show the mean (error bars denote SEM) of three independent biological replicates. (**C–J**)THP-1 cells were infected with HIV-1_NL4-3_/VSV-G at an MOI of 5 i.u./cell (titered on TZM-bL cells) in the absence or presence of GS-CA1 (10, 25, or 100 nM), lenacapavir (10, 25, or 100 nM) or nevirapine (NVP, 25 µM). ISG (*IFIT2*, *ISG15*, *MX1*, *CXCL10*) expression (**C–F**), early RT products (**G**), late RT products (**H**), 2-LTRs (**I**), and integration (Alu-PCR, **J**) were analyzed by qPCR at 24 hpi. Data in C–F are normalized relative to uninfected and mock-treated cells. Data in G–I are normalized relative to HIV-1-infected and mock-treated cells (set to 1). Data are derived from three independent biological replicates. Graphs show the mean with error bars displaying the SEM.

Lenacapavir (GS-6207) is a highly potent CA-targeting compound that impacts multiple steps of virus replication in a dose-dependent manner (70, 71). Lenacapavir and the related compound GS-CA1 have been shown to enhance the stability of the CA lattice *in vitro* and in cells by promoting distal intra- and inter-hexamer interactions, possibly resulting in fractures within the CA lattice (54, 70, 72–74). To validate the impact of GS-CA1 and lenacapavir on core stability in infected cells, we first conducted “fate of core” assays tracking CA and RT products. At 100 nM, both compounds enhanced core-associated CA in denser fractions and reduced soluble CA (Fig. 2A) but eliminated the accumulation of RT products in dense fractions (Fig. 2B). At lower concentrations, effects on core-associated CA levels were expectedly more modest, but we noted a dose-dependent reduction in the accumulation of RT products (Fig. S2A and B).

At 100 nM, both GS-CA1 and lenacapavir blocked ISG induction at levels similar to the RT inhibitor nevirapine (NVP) (Fig. 2C through F), most likely as a result of reverse transcription inhibition (Fig. 2G and H). At 25 nM, although lenacapavir reduced reverse transcription at levels similar to treatment with NVP (Fig. 2G and H; Fig. S2B), ISG expression was still induced (Fig. 2C through F), albeit fourfold to fivefold lower than mock-treated cells. At 10 nM, despite a reduction in the accumulation of RT products (Fig. 2G and H; Fig. S2B), lenacapavir induced ISG expression at levels similar to mock-treated cells (Fig. 2C through F). GS-CA1 had more modest effects on reverse transcription at these lower concentrations (Fig. 2G and H; Fig. S2B) but resulted in ISG induction at levels similar to that observed in mock-treated cells (Fig. 2C through F). In line with prior findings (70–72), at 10 nM and 25 nM, both GS-CA1 and lenacapavir more potently reduced the accumulation of 2-LTR circles (marking nuclear entry) and integration compared to reverse transcription (Fig. 2I and J), demonstrating the multi-modal action of these compounds.

We next tested whether lower doses of lenacapavir that block nuclear entry, but not reverse transcription, affect innate immune activation by WT HIV-1. At 3 nM, lenacapavir modestly enhanced ISG induction (Fig. S2C through E), albeit this difference remained statistically insignificant. In addition, we found a dose-dependent decrease in ISG expression with lower concentrations of lenacapavir (Fig. S2C through E). Expectedly, at this concentration range, lenacapavir did not reduce RT product accumulation (Fig. S2F and G) but inhibited the accumulation of 2-LTR circles in a dose-dependent manner (Fig. S2H). Taken together, these findings suggest that both GS-CA1 and lenacapavir can modestly enhance the sensing of viral RT products through modulation of CA stability and to a lesser extent by trafficking of RT products.

### Genetically altering HIV-1 capsid lattice stability impacts innate immune sensing

Several CA substitutions that affect inter- and intra-hexamer interactions have well-defined, biochemically tractable effects on the stability of the CA lattice (59, 62, 69). For example, P38A, K203A, and Q219A substitutions decrease stability, whereas the E45A substitution enhances the stability of the CA lattice both *in vitro* and in cells (59, 62, 69). We have previously shown that destabilization of the CA lattice by P38A, K203A, and Q219A substitutions results in premature loss of the incoming genomic RNA (gRNA), in part explaining the well-described reverse transcription defect of these viruses (69). Hence, we reasoned that loss of CA lattice stability may result in the sensing of exposed gRNA (prior to degradation) or RT products, provided that CA lattice integrity is maintained long enough post-entry to allow reverse transcription.

**Fig 3:**
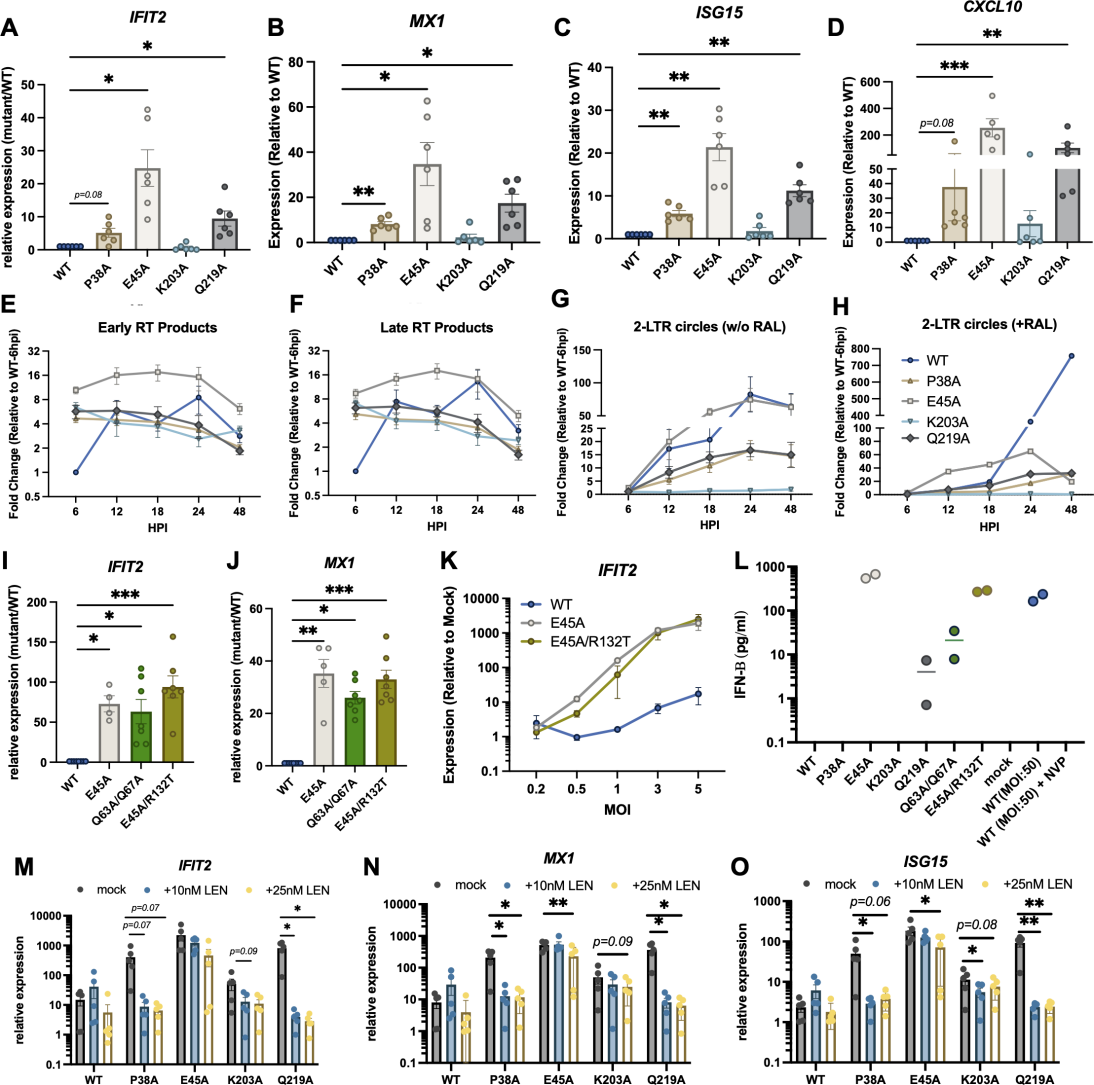
CA-destabilizing and -stabilizing mutations affect innate immune sensing of HIV-1. (**A–J**) THP-1 cells were infected with HIV-1_NL4-3_/VSV-G at an MOI of 5 i.u./cell (titered on TZM-bL cells) or an equivalent particle number of CA mutants (normalized by RT activity). A parallel set of samples were infected in the presence of 10 µM raltegravir (**H**). qPCR analysis of ISG (*IFIT2*, *MX1*, *ISG15*, and *CXCL10*) expression at 24 hpi (**A–D, I, J**), early RT products (**E**), late RT products (**F**), and 2-LTR circles in the absence (**G**) and presence of raltegravir (**H**) at the indicated time points is shown. ISG expression in A–D, I, and J is normalized relative to cells infected with WT HIV-1 (set to 1). Data in E–H are normalized relative to WT HIV-1-infected cells at 6hpi (set to 1). Graphs show the mean derived from 4 to 7 (**A–D, I, J**), 5 (**E–G**), and 2 (**H**) independent biological replicates with error bars displaying the SEM (*P < 0.05; **P < 0.01; ***P < 0.001, by one-way ANOVA multiple comparison test with Dunnett’s correction). (**K**) THP-1 cells were infected with HIV-1_NL4-3_/VSV-G at the indicated MOIs or an equivalent particle number of the CA mutants. *IFIT2* expression was analyzed at 24 hpi by qPCR and normalized relative to mock-infected cells. Data show the mean from two independent biological replicates, error bars show the SEM. (**L**) Cell culture supernatants collected from infected THP-1 cells were subjected to an IFN-β ELISA assay as detailed in Materials and Methods (*n* = 2 biological replicates). (**M–O**) THP-1 cells were infected as above in the absence or presence of 10 nM or 25 nM lenacapavir. The expression of *IFIT2* (**M**), *MX1* (**N**), and *ISG15* (**O**) was analyzed at 24 hpi by qPCR and normalized relative to mock-infected/mock-treated cells. Graphs show the mean derived from five independent replicates whereby error bars denote the SEM (*P < 0.05; **P < 0.01, by paired *t*-test with Holm-Sidak correction).

We infected THP-1 cells with equivalent particle numbers (based on RT activity in virions) of WT and CA mutant viruses and assessed ISG induction. We found that CA lattice-destabilizing substitutions P38A and Q219A both induced higher levels of innate immune activation (Fig. 3A through D) that was fully dependent on reverse transcription (Fig. S3A). This enhanced innate immune activation was observed despite a notable decrease in the accumulation of RT products and 2-LTR circles that mark nuclear entry at later time points in infection (Fig. 3E through H). By contrast, the CA K203A-destabilizing substitution did not induce higher levels of ISGs (Fig. 3A through D; Fig. S3A), likely due to a more severe impairment of reverse transcription (Fig. 3E through H). Surprisingly, viruses containing the CA lattice-stabilizing E45A substitution induced ~20-fold higher levels of ISG expression compared to WT HIV-1 (Fig. 3A through D; Fig. S3A). This enhancement correlated with the HIV-1 CA E45A virus accumulating significantly higher levels of RT products throughout the infection (Fig. 3E and F) and a nuclear entry defect (67, 75), as evident in the lower accumulation of 2-LTR circles in the presence of integrase strand transfer inhibitor raltegravir (Fig. 3H). Importantly, viral stocks had similar ratios of gRNA, RT, and CA, and as such it is unlikely that the observed differences in innate immune activation between CA mutants are due to RT activity-based normalization of input virus (Fig. S3B). Furthermore, the heightened ISG induction with the CA mutants, in particular, E45A, was also evident at lower MOIs (Fig. S3C). Despite the differences in the kinetics of RT product accumulation between the CA mutants (Fig. 3E and F), ISG induction kinetics was largely similar and remained high for P38A, E45A, and Q219A mutants across all time points (Fig. S3D through F).

Similar to CA E45A, CA Q63A/Q67A mutant cores have been reported to disassemble with slower kinetics than WT cores in cell-based assays (45, 68, 76, 77). HIV-1 CA Q63A/Q67A displayed a similar phenotype to CA E45A in THP-1 cells and accumulated higher than WT levels of RT products (Fig. S3G and H), but a >10-fold defect in the accumulation of 2-LTR circles suggested a nuclear entry block (Fig. S3I). HIV-1 CA Q63A/Q67A induced innate immune activation at levels significantly higher than HIV-1 WT and nearing that induced by the CA E45A mutant (Fig. 3I and J; Fig. S3J). The CA R132T substitution has been reported to suppress the replication defect of the CA E45A mutant in part by overcoming its nuclear entry defect (75). In agreement, HIV-1 CA E45A/R132T accumulated similarly high levels of RT products compared to WT HIV-1 (Fig. S3G and H) but displayed a marked increase in 2-LTR circles over CA E45A (Fig. S3I). Despite enhanced nuclear entry, CA E45A/R132T induced ISG expression at significantly higher levels than WT HIV-1 (Fig. 3I and J; Fig. S3J). On the other hand, compared to CA E45A, CA E45A/R132T induced modestly less (2–3-fold) *IFIT2* expression though this effect was only apparent at lower MOIs (Fig. 3K). For all CA mutants, the degree of ISG induction correlated with IFN-β protein secretion in cell culture supernatants (Fig. 3L). Notably, IFN-β secretion in cells infected with HIV-1 WT, CA P38A, or CA K203A mutants remained below the limit of detection (Fig. 3L), in line with the comparably low levels of innate immune activation induced with these mutants. In addition, a high MOI infection with WT HIV-1 (MOI: 50 i.u./cell) resulted in a significant reverse transcription-dependent increase in IFN-β protein secretion in cell culture supernatants (Fig. 3L).

CA-destabilizing P38A, K203A, and Q219A substitutions have recently been shown to confer increased sensitivity to lenacapavir (74). We next determined the combined effects of CA destabilization and lenacapavir treatment on innate immune activation. Interestingly, despite similar levels of reduction in RT product and 2-LTR circle accumulation compared to WT (Fig. S3K through M), the enhanced ISG expression observed with the P38A and Q219A mutants was completely abolished with 10 nM lenacapavir, whereas WT HIV-1 and the CA E45A mutant remained insensitive (Fig. 3M through O). Taken together, these results demonstrate that decreasing CA lattice stability can result in more potent innate immune sensing of HIV-1 RT products, which can be counteracted by the stabilizing effects of lenacapavir at low concentrations. The unexpected increase in innate immune sensing with the hyperstable CA mutants can be ascribed to the elevated levels and faster kinetics of reverse transcription in the cytosol, whereas cytosolic accumulation of the RT products *per se* may not play a big role.

### Innate immune sensing of HIV-1 reverse transcription products is dependent on the cGAS-STING pathway

**Fig 4:**
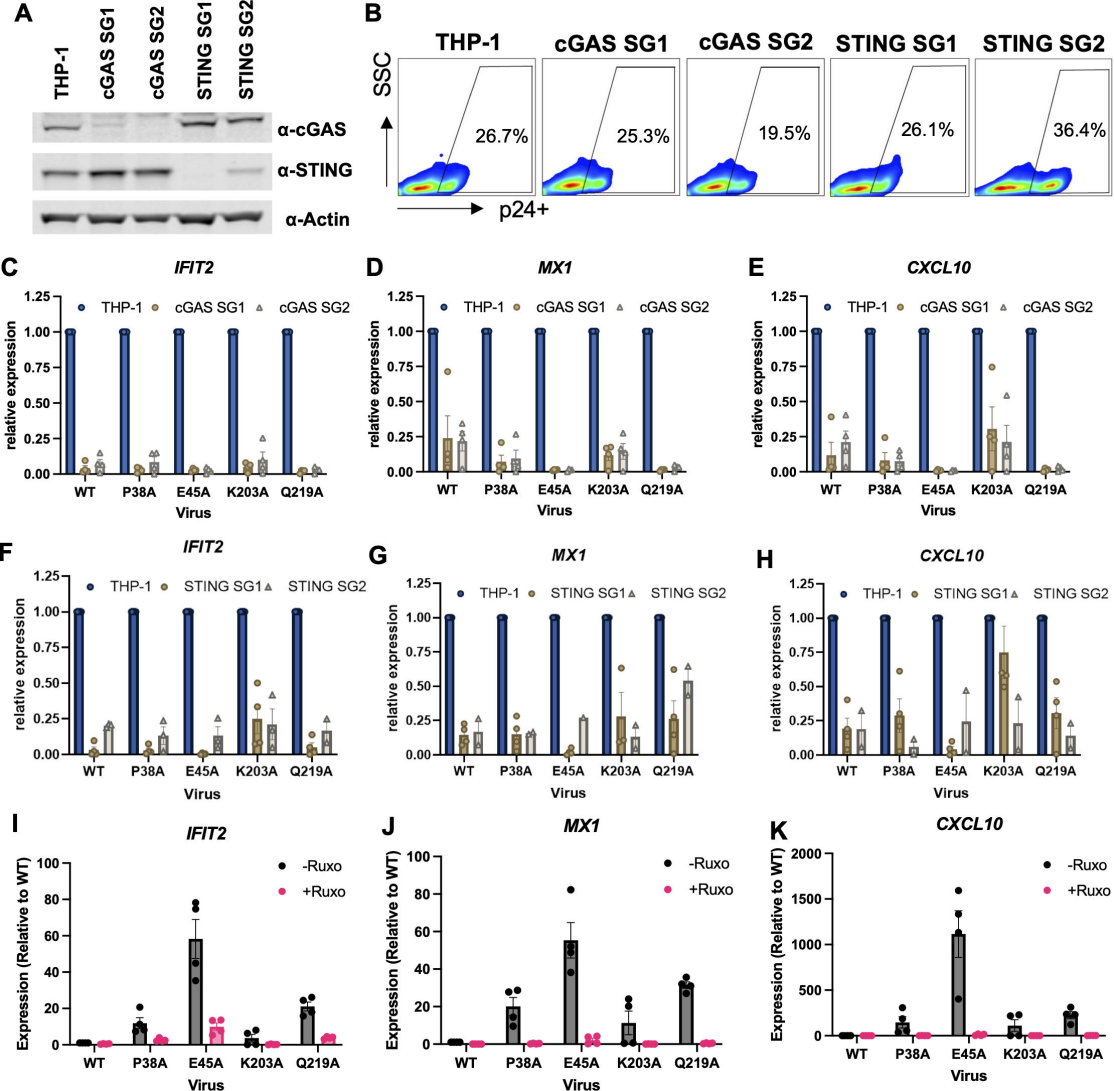
Innate sensing of WT HIV-1 and CA mutants is mediated by the cGAS-STING pathway. (**A**) THP-1 cells were subjected to CRISPR-mediated knockout of cGAS and STING using two different guides (SG1 and SG2) per target. Bulk populations of the indicated cells were analyzed by immunoblotting for cGAS, STING, and actin (loading control). (**B**) Parental THP-1, cGAS, and STING knockout cells were infected with HIV-1_NL4-3_/VSV-G at an MOI of 5 i.u./cell (titered on TZM-bL cells). Cells were fixed at 48 hpi and the rate of infection was analyzed by CA-p24 staining and flow cytometry. (**C–H**) Parental THP-1, cGAS, and STING knockout cells were infected with HIV-1_NL4-3_/VSV-G at an MOI of 5 i.u./cell (titered on TZM-bL cells) or an equivalent particle number of CA mutants (normalized by RT activity). ISG (*IFIT2*, *MX1*, and *CXCL10*) expression was analyzed by qRT-PCR at 24 hpi and normalized relative to non-transduced THP-1 cells for each virus (set to 1) to display the impact of cGAS-STING knockout. Graphs show the mean derived from 2 to 4 independent biological replicates and error bars display the SEM. (**I–K**) THP-1 cells were infected as above in the presence of 1 µM ruxolitinib. ISG (*IFIT2*, *MX1*, and *CXCL10*) expression was analyzed by qRT-PCR at 24 hpi and normalized relative to mock-treated cells infected with WT HIV-1 (set to 1). Graphs show the mean derived from four independent biological replicates with error bars displaying the SEM.

We next investigated whether the observed differences in ISG induction by the CA mutants are mediated by type-I IFN signaling by assessing the involvement of different nucleotide sensing pathways. As expected from the dependence of innate immune activation on reverse transcription, we found that CRISPR-mediated knockout of cytosolic DNA sensors cGAS and STING (Fig. 4A) substantially reduced ISG induction (Fig. 4C through H) without affecting infection rates (Fig. 4B) or accumulation of RT products (data not shown). Other DNA sensors, including IFI16 and CA-binding adaptor proteins PQBP1 and NONO, did not appear to be involved in sensing of WT HIV-1 or viruses bearing CA mutations (Fig. S4A through I). Predictably, knockout of TREX1 increased ISG induction with WT and all CA mutant viruses (Fig. S4J through L). Furthermore, we found no significant contribution of RNA-sensing pathways to innate immune sensing of HIV-1, as knockout of key adaptors MyD88 and MAVS, which act downstream of TLR and RLR sensing pathways, respectively, did not reduce innate immune activation (Fig. S4M through O). Of note, all knockout cells were infected at similar levels as control cells (Fig. S4B, E, H, K, and N), suggesting that the observed impact (or its lack thereof) cannot be ascribed to different rates of infection. Finally, infections done in the presence of the JAK1/2 inhibitor ruxolitinib substantially decreased ISG expression for WT CA as well as CA mutants (Fig. 4I through K), suggesting the involvement of the canonical type I IFN pathway upon cGAS-mediated sensing. Overall, these findings demonstrate that, similar to WT HIV-1, CA stability mutants are sensed by cGAS-STING, without any evidence of innate immune activation mediated by RNA-sensing pathways.

### Cytosolic accumulation of RT products contributes to potent sensing of the hyperstable CA mutants

**Fig 5:**
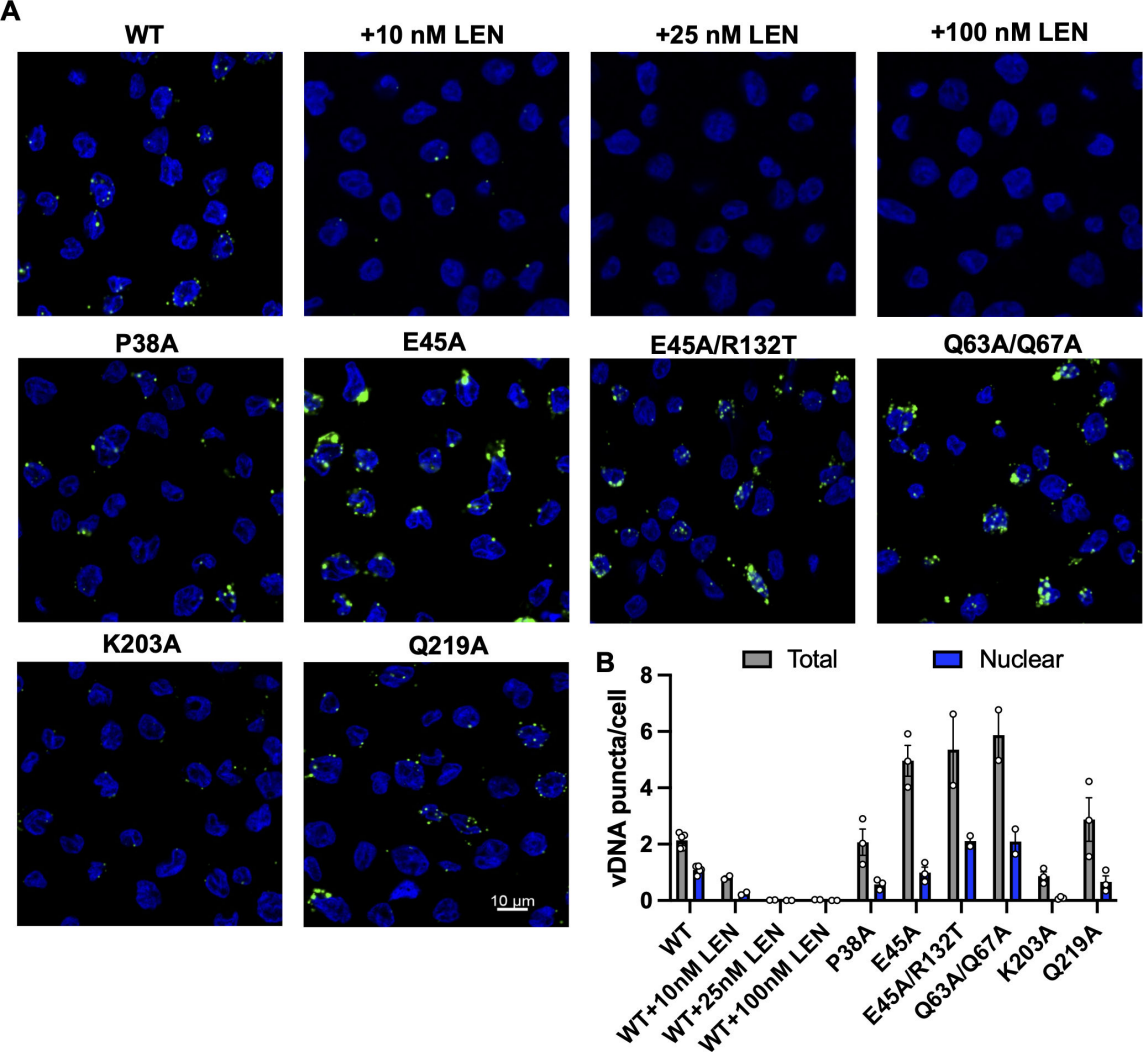
Accumulation and localization of RT products in infected cells. (**A**) Representative images of THP-1 cells infected with WT HIV-1_NL4-3_/VSV-G (as in above the figures) in the presence of 10, 25, or 100 nM lenacapavir (LEN) or the indicated CA mutant viruses at 24 hpi. Cells were stained for RT products by FISH (green) and nuclei by DAPI (blue) as detailed in Materials and Methods. (**B**) Quantitation of total and nuclear RT products per cell as detailed in Materials and Methods. The graph shows the mean derived from two to three independent biological replicates. Error bars display the SEM.

In addition to providing physical protection for incoming vRNPs to allow reverse transcription, CA is also a key determinant of nuclear entry (67, 77–85), which may influence the observed differences in innate immune activation by CA mutants and CA-targeting compounds. To test this, we analyzed the subcellular localization of RT products upon altering CA stability. In agreement with the qPCR data (Fig. 2G through J), 10 nM lenacapavir reduced both the synthesis and nuclear localization of RT products, whereas higher concentrations (25 nM and 100 nM) strongly inhibited RT product synthesis (Fig. 5A and B; Fig. S5A). Compared to WT HIV-1, the P38A and Q219A mutants appeared to accumulate similar levels of RT products per cell, but a proportional decrease in nuclear localization was observed (Fig. 5A and B; Fig. S5A). Hence, the enhanced immune activation observed with these CA mutants is likely due to their destabilizing effects on the CA lattice. As expected, the K203A mutant displayed a more severe impairment of reverse transcription (Fig. 5A and B; Fig. S5A). In agreement with the qPCR data (Fig. S3G and H), infection with the hyperstable CA E45A and Q63A/Q67A mutants resulted in substantially higher level of RT product accumulation; however, a greater proportion of these products localized in the cytosol (Fig. 5A and B; Fig. S5A). Although HIV-1 CA E45A/R132T measurably enhanced nuclear entry of the reverse-transcribed viral DNA compared to the CA E45A mutant, RT products still accumulated to high levels compared to WT HIV-1 (Fig. 5A and B; Fig. S5A), likely explaining the high level of innate immune activation observed with this mutant (Fig. 3I through K; Fig. S3J). As expected from the defects in RT product and 2-LTR circle accumulation (Fig. 3E through H; Fig. S3G through I), exception WT CA and CA E45A/R132T, all other CA mutants yielded lower integrated proviruses (Fig. S5B). Overall, these findings suggest that in addition to CA stability, cytosolic accumulation of the RT products may also contribute to innate sensing of HIV-1 replication.

### Impact of CA stability on innate immune sensing in primary macrophages

**Fig 6:**
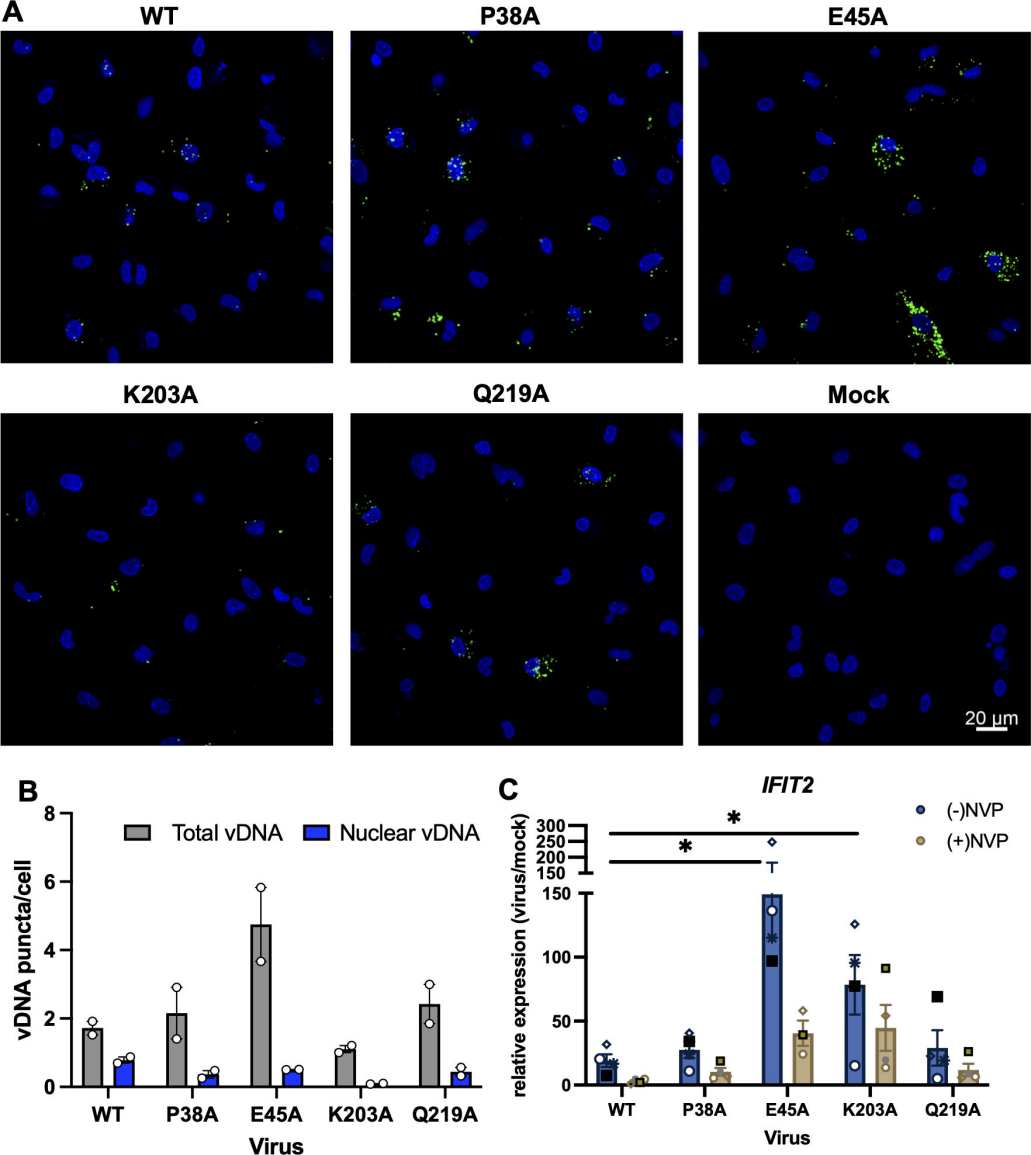
CA mutations affect localization and sensing of RT products in human MDMs. (**A**) Representative images of human MDMs infected with WT HIV-1_NL4-3_/VSV-G at an MOI of 5 i.u./cell (titered on TZM-bL cells) or an equivalent particle number of CA mutants (normalized by RT activity) at 12 hpi. Cells were stained for RT products by FISH (green) and nuclei DAPI (blue) as detailed in Materials and Methods. (**B**) Quantitation of total and nuclear RT products per cell. The graph shows the mean derived from independent biological replicates from two donors. Error bars display the SEM. (**C**) Human MDMs were infected with HIV-1_NL4-3_/VSV-G at an MOI of 5 i.u./cell (titered on TZM-bL cells) or an equivalent particle number of CA mutants (normalized by RT activity) in the absence or presence of 25 µM NVP. *IFIT2* expression was analyzed by qPCR at 24hpi and normalized relative to mock-infected cells. Graphs show the mean derived from independent biological replicates from four donors and error bars display the SEM.

We next tested whether tampering with CA stability leads to similar differences in innate immune activation in primary monocyte-derived macrophages (MDMs). As in THP-1 cells, we found that viruses bearing the P38A- and Q219A-destabilizing mutations accumulated similar levels of RT products as WT, whereas the HIV-1 CA K203A was significantly more impaired (Fig. 6A and B; Fig. S6). CA E45A mutant virus accumulated greater levels of RT products; however, as in THP-1 cells, a greater fraction of them accumulated in the cytosol (Fig. 6A and B; Fig. S6). A similar decrease in the localization of RT products in the nucleus was observed for P38A and Q219A mutants (Fig. 6A and B; Fig. S6). Expectedly, the E45A mutant induced significantly higher levels of innate immune activation in primary MDMs, whereas P38A and Q219A mutants had more modest effects (Fig. 6C). Interestingly, in contrast to findings in THP-1 cells, we found that the K203A mutant induced higher levels of ISGs, yet this induction was largely independent of reverse transcription (Fig. 6C), raising the possibility of a separate pathway involved in its sensing. Overall, in agreement with results from THP-1 cells, our findings demonstrate that altering CA stability and subcellular localization of RT products can result in more potent induction of innate immune responses in primary macrophages.

## DISCUSSION

In this study, we utilized chemical and genetic approaches to determine whether direct manipulation of HIV-1 CA lattice stability affects innate immune sensing of viral nucleic acids. We found that decreasing the stability of the CA lattice *via* the P38A and Q219A substitutions resulted in more potent sensing of RT products. While the CA K203A-derived cores are biochemically indistinguishable from P38A and Q219A *in vitro* and in cell-based assays (69), we found that K203A mutation led to more prominent reverse transcription defects and, likely as a result, did not induce heightened innate immune activation in THP-1 cells.

A counterintuitive finding in our study was that enhancing core stability *via* the E45A and Q63A/Q67A substitutions resulted in substantially greater levels of innate immune activation, which we ascribe to the combined effects of enhanced RT product synthesis, their cytosolic accumulation, and possibly the enhanced core permeability previously reported with the CA E45A mutant (86). Partial rescue of the CA E45A nuclear entry defect with CA E45A/R132T double mutant resulted in only modestly lower levels of innate immune activation, suggesting that the main factors that contribute to sensing of HIV-1 CA E45A are the high levels of RT product accumulation combined with effects on CA permeability rather than the nuclear entry block. One caveat is that the R132T mutation only partially rescues the infectivity defect of E45A, possibly masking the impact of cytosolic DNA accumulation on innate sensing. In-depth studies will be needed to parse out the effects of nuclear entry block on innate immune activation. Overall, findings from CA mutants were recapitulated in primary MDMs, except the K203A substitution that induced higher than WT levels of ISG expression, despite lower levels of RT product accumulation, suggesting the involvement of another innate sensing pathway.

Despite recent studies that suggested that conical cores protect RT complexes up to the point of nuclear entry (49–55), our findings raise the possibility that RT products must be accessible to cGAS even under conditions that supposedly stabilize the CA lattice. The low levels of innate immune activation by WT HIV-1 compared to the CA E45A mutant, which is both competent for reverse transcription, are possibly due to a combination of slower RT kinetics, completion of reverse transcription in the nucleus, more complete protection with CA and faster transit of RTC complexes in the cytosol. Of note, WT HIV-1 can result in a similar degree of innate immune activation as the CA E45A mutant in a manner dependent on reverse transcription at substantially higher MOIs, suggesting that it is capable of innate immune activation despite CA protection. Although more advanced single-cell studies are needed to determine how many copies of cytosolic vDNA are needed to activate cGAS, these results suggest that a single infectious virus is unlikely to trigger innate immune activation in cells. One possible complication of high MOI infections is that we cannot rule out the presence of low levels of noninfectious virus particles that may be competent for reverse transcription but are blocked at a stage downstream from it. Alternatively, the possibly limiting nature of reverse transcription and nuclear entry may result in the accumulation of incomplete RT products in the cytosol that are more prone to sensing.

The small molecule CA inhibitor lenacapavir is a promising, long-acting antiretroviral that blocks multiple functions of CA in a dose-dependent manner (54, 70, 72, 73, 87). We found that at low concentrations of lenacapavir and GS-CA1 that allow accumulation of RT products (albeit lower than mock-treated cells), WT HIV-1 was still able to induce innate immune activation. This observation is in line with the findings that GS-CA1 and lenacapavir increase the stability of the CA lattice and subsequently cause fractures within the lattice that may allow access of replication intermediates to innate immune sensors (88). The inability of WT HIV-1 to induce innate immune activation to the same extent as the CA mutants in the presence of lenacapavir can be explained by the incomplete fractures within the CA lattice that provide some degree of protection from cGAS. Whether or not the findings with lenacapavir will extend to immune activation and impact T-cell exhaustion *in vivo* remains to be determined.

Several studies have demonstrated that HIV-1 can successfully evade sensing and activation of innate immunity, whereas others have provided evidence that even WT HIV-1 replication can be sensed (18, 33, 35–38). This variation in sensing can be explained by differences in cell culture conditions, virus production, and MOI. For example, we used unstimulated THP-1 cells in our studies, as we found that stimulation of THP-1 cells with phorbol-12-myristate-13-acetate results in lower rates of infection (18), necessitating comparably larger virus inoculums that can have a big impact in innate immune sensing due to carry over of contaminants (38). We also followed published protocols to minimize contamination of virus stocks by plasmids and LPS, which may contribute to innate immune activation (38). Although virus inoculum volumes were different between CA mutants, which is unavoidable due to normalization based on particle numbers, we did not observe a trend with larger virus volumes resulting in higher levels of ISG induction. In addition, the consistent patterns observed with mutations that stabilize vs destabilize the CA lattice also engender the confidence that the observed differences in innate immune activation are due to CA-related outcomes. Above all, the dependence of the innate immune activation by WT HIV-1 and CA variants thereof on reverse transcription and lack of involvement of MyD88 argues that these contaminants are unlikely to contribute to sensing in the presented experimental setup.

Myeloid cells express both DNA- and RNA-sensing pathway components that can presumably be activated during early post-entry stages of HIV-1 infection. Hence, it is intriguing that only the RT products are sensed *via* the cGAS-STING pathway, with no involvement of RNA-sensing pathways upon destabilization of the CA lattice. There are a few possibilities that may underlie the lack of sensing of incoming vRNA in myeloid cells. First, we previously described that CA destabilization leads to faster disassembly of the lattice and premature loss of the vRNA genome in target cells (69). Second, RT products may be more accessible to host cell sensors, as reverse transcription may facilitate uncoating (41–48). Third, incoming vRNA may be nicked and deadenylated in virions (89–91), and lack structural features that may be required for sensing by RLRs. Lastly, innate immune activation in myeloid cells by intron-containing vRNAs may require their tethering to the cell plasma membrane (30) and hence it is unlikely that incoming vRNAs can be sensed through this pathway. One exception is the CA K203A mutant, which induces near equivalent levels of ISG expression despite RT inhibition in MDMs and suggests that RNA-sensing pathways may be more dominant in this setting. In addition, despite a significant reduction in ISG expression in response to nevirapine, the CA E45A mutant still induced significant ISG expression indicating that both DNA and RNA sensing pathways contribute to its sensing. It remains to be determined whether sensing in these cases is mediated by similar mechanisms that act on intron-containing viral RNAs (29, 30) or canonical RNA-sensing pathways.

In addition to cellular recognition of viral nucleic acids, recent studies have suggested the involvement of accessory host factors that potentiate cGAS-dependent sensing. For example, PQBP1 has been described as an adapter that aids in the recruitment of cGAS to CA and the subsequent initiation of immune signaling (26, 27). In our experimental setup, PQBP1 did not seem to be involved in cGAS-mediated sensing, suggesting that its involvement could depend on the context of infection. Another cellular factor that has been implicated in the recognition of CA is NONO, which potentiates cGAS-mediated sensing by recognizing nuclear CA in monocyte-derived dendritic cells and macrophages (28). However, given the nuclear entry defects of CA mutants, it is unlikely that NONO contributes to the more potent innate immune activation observed with these viruses, as also supported by our knockout studies.

Although we ascribed the more potent innate immune activation observed by CA mutants to direct effects on capsid lattice stability and subcellular localization of RT products, we cannot exclude the possibility that these mutations may have additionally impacted interactions of CA by other known and unknown host co-factors. For example, others have shown that CA mutations that prevent the recruitment of cellular cofactors CPSF6 and cyclophilin A can enhance innate immune sensing (14) and viruses that are unable to properly package IP6 are sensed more potently *via* the cGAS-STING pathway (39). Importantly, CA P38A and Q219A viruses require IP6 for reverse transcription (74), and this dependency may also contribute to their more potent innate immune sensing.

HIV-1 utilizes the host cell’s microtubule network to traffic through the cytoplasm and enter the nucleus (92–95). One hypothesis that accounts for the accumulation of RT products in the cytosol is that the mutations that alter CA stability block interaction with important trafficking molecules or other steps at nuclear entry, as has been suggested for the E45A mutant (67, 68, 87). Although enhancing CA stability with the E45A mutant (and possibly CA targeting compounds) can allow for the synthesis of more RT products and hence may be a favorable outcome, the trade-off of this enhanced stability in these few instances appears to be defects in nuclear entry and increased sensitivity to innate sensing.

In summary, our study revealed that directly modifying CA stability can boost the recognition of HIV-1 reverse transcription products through the cGAS-STING pathway. Given the multiple roles of CA in the early stages of infection, it will be important to tease apart which function is more critical for innate sensing. It is noteworthy that in none of the conditions tested in this study, we observed lower than WT levels of innate immune induction, except for the combination of CA destabilizing mutations with the CA lattice stabilizing compound lenacapavir. Thus, we propose that HIV-1 CA stability, reverse transcription, and trafficking of these RT products in the cytosol are finely balanced to minimize sensing of RT products *via* the cGAS-STING pathway.

## MATERIALS AND METHODS

### Cell lines, primary cells, and compounds

HEK293T cells (ATCC CRL-11268) and HeLa-derived TZM-bL cells (NIH AIDS Reagent Program) were maintained in Dulbecco’s modified Eagle’s medium supplemented with 10% fetal bovine serum. CHO K1-derived pgsA-745 cells (ATCC CRL-2242) were maintained in Dulbecco’s modified Eagle/F12 media supplemented with 10% fetal bovine serum. THP-1 cells (ATCC#TIB-202) were grown in RPMI-1640 media supplemented with 10% heat-inactivated fetal bovine serum.

Anonymous peripheral blood samples were acquired from the Mississippi Valley Regional Blood Center. Peripheral blood mononuclear cells (PBMCs) were isolated using Ficoll and SepMate PBMC isolation tubes (STEMCELL #8450). Human CD14+ cells were isolated using MojoSort Human CD14 Selection Kit (Biolegend #480026) from PBMCs. To generate human monocyte-derived macrophages (MDMs), purified monocytes were stimulated with 50 ng/mL M-CSF (Biolegend #574806) and 50 ng/mL GM-CSF (Biolegend #572904) in RPMI 1640 Medium containing 10% fetal bovine serum and Penicillin-Streptomycin for 6 days. Fresh media containing GM-CSF and M-CSF was provided without disturbing the attached cells on day 3. At day 6, the differentiated macrophages were treated with Accutase (Biolegend #423201), re-plated in 48 wells, and rested overnight before infection.

Lentiviral particles for CRISPR were produced by polyethyleneimine-mediated transfection of six-well plates of HEK293T cells with 1 µg of pLentiCRISPRv2 puro (Addgene #98290), encoding gene-specific guide RNAs, 1 µg of PSPAX packaging plasmid, and 0.2 µg of VSV-G expression plasmid. Virus supernatants were collected at 48 h post-transfection, passed through 0.2 µm filters, and used to transduce THP-1 cells by spinoculation (800 × *g,* 45 min, 32°C). Bulk populations of transduced cells were selected using puromycin (2 µg/mL). Cell lines were screened for successful gene knockout by immunoblotting and Sanger sequencing. Cell lines were maintained in RPMI media supplemented with 10% heat-inactivated fetal bovine serum and 2 µg/mL puromycin.

GS-CA1 and GS-6207 (lenacapavir) were kindly provided by Gilead Sciences and Mamuka Kvaratskhelia, respectively. The preparation of GS-6207 was explained before (72). Compounds were prepared in dimethyl sulfoxide and stored at −80°C. Nevirapine and raltegravir were obtained from NIH AIDS Reagents.

### Viruses and infections

Vesicular stomatitis virus G protein (VSV-G)-pseudotyped viruses were produced by transfection of HEK293T cells with pNL4-3-derived plasmids and VSV-G expression plasmid at a ratio of 9:1, respectively, using polyethyleneimine (PolySciences). Taking into account a recent report that suggested that LPS carryover from plasmids can misleadingly potentiate cGAS-mediated sensing in infected cells (38), we made sure to include an endotoxin wash in plasmid preparations and change media in virus-producing cells that together minimizes LPS contamination. In addition, we made sure to repeat all critical experiments with independently prepared plasmids as well as virus stocks. Cell culture supernatants were collected 48 h post-transfection, filtered, treated with DNase I, and concentrated 10-fold with Lenti-X Concentrator (Takara Bio).

On the date of infection, 1 × 10^5^ THP-1 cells were plated in media containing 5 µg/mL polybrene. Cells were infected with VSV-G-pseudotyped viruses at an MOI of 5. Viral titers were determined on HeLa-derived TZM-bL reporter cells. Viruses bearing CA mutations were normalized based on RT activity by a qPCR-based assay in most experiments. As described above, MDMs were plated the day before infection and infected in the presence of 5 µg/mL polybrene.

### Plasmids

Mutations in the CA coding sequence were introduced into the full-length replication competent pNL4-3 by overlap extension PCR as described before (69). Briefly, forward and reverse primers containing CA mutations were used in PCRs with antisense and sense outer primers containing unique restriction endonuclease sites (SphI-sense-AgeI-antisense, respectively). The resulting fragments containing CA mutations were mixed at a 1:1 ratio and overlapped subsequently using the outer sense and antisense primer pairs. PCR products were digested with the corresponding restriction endonucleases and ligated with appropriately digested pNL4-3 plasmid vector fragments. The presence of engineered mutations and lack of unwanted extraneous mutations were verified by Sanger sequencing.

### CRISPR sgRNA cloning scheme

Target sequences were obtained from CRISPick (Broad Institute). The pLentiCRISPRv2 plasmid (Addgene ID #98290) was digested with BsmBI for 2 h at 55°C. Designed oligos (Table 1) were phosphorylated with T4 PNK at 37°C for 30 min and annealed. The resulting DNA was diluted 1:200 with H_2_O, ligated to the digested vector, and transformed into DH10B cells. The presence of the designed sgRNA oligos was verified by sequencing.

**TABLE 1:** sgRNA sequences used in targeting of the indicated host factors.

Target	Sequence
CGAS	ATCCCTCCGTACGAGAATGG
CGAS	AGACTCGGTGGGATCCATCG
IFI16	AAGCAGCAGTTTCTTAACCA
IFI16	CAAGAGCATGAAGCTACCCC
MAVS	AGCCTATCATCTGCTCCAGT
MAVS	ACTGGAGCAGATGATAGGCT
MYD88	CAGCGACATCCAGTTTGTGC
MYD88	ACCACACTTGATGACCCCCT
PQBP1	GGGCCACGACAAGTCTGACA
PQBP1	AGCCATGAGAAACTAGACAG
STING	CATATTACATCGGATATCTG
STING	CATTACAACAACCTGCTACG
TREX1	GTCCCCTCCAGACTCGCACA
TREX1	TCTGGATGGTGCCTTCTGTG
NONO	GCTCTGGACAGATGCAGTGA
NONO	GACCAGTTAGATGATGAAGA

### Immunoblotting

Cells lysed in 1× RIPA buffer were resuspended in SDS sample buffer, separated by electrophoresis on Bolt 4%–12% Bis-Tris Plus gels (Invitrogen), blotted onto nitrocellulose membranes, and probed with the following antibodies: mouse monoclonal anti-p24 (CA) antibody (183-H12-5C; NIH AIDS reagents), mouse monoclonal anti-Actin antibody (Santa Cruz Biotechnology #sc-8432), the rabbit polyclonal anti-PQBP1 antibody (Bethyl Laboratories #A302-802A), or the following rabbit monoclonal antibodies: anti-cGAS (Cell Signaling Technology #15102S), anti-IFI16 (Cell Signaling Technology #14970S), anti-MAVS (Cell Signaling Technology #24930S), anti-MyD88 (Cell Signaling Technology #4283S), anti-STING (Cell Signaling Technology #13647S), anti-NONO (Millipore Sigma, #05–950) and anti-TREX1 (Cell Signaling Technology #15107S).

### RNA extraction and qRT-PCR

Infected cells were collected at 6, 12, 18, 24, or 48 hpi, and RNA was extracted by TRIzol according to the manufacturer’s instructions. The resulting RNA was reverse transcribed with a high-capacity reverse transcription kit (Applied Biosystems #4368813) and subjected to SYBR-green-based qPCR analysis for ISG expression using the below primer pairs (Table 2).

**TABLE 2:** Primers used to detect ISGs and 18S rRNA in qRT-PCR assays.

Target	Sequence
*IFIT2*	Fwd 5′-AGCTGAGAATTGCACTGCAACCATG-3′ Rev 5′-CTCCATCAAGTTCCAGGTGAAATGGC-3′
*MX1*	Fwd 5′-CACTGCGAGGAGATCGGTTC-3′ Rev 5′-CTGTTCTCCTGCACCTCCTTG-3′
*ISG15*	Fwd 5′- CACAGCCCACAGCCCACAG-3′ Rev 5′-GCTCAGGGACACCTGGAATTCG-3′
*CXCL10*	Fwd 5′-AGCAGTTAGCAAGGAAAGGTCT-3′ Rev 5′-GCCTCTGTGTGGTCCATCCT-3′
*SIGLEC1*	Fwd 5′-CCTCGGGGAGGAACATCCTT-3′ Rev 5′-AGGCGTACCCCATCCTTGA-3′
*18S rRNA*	Fwd 5′-CCGCAGCTAGGAATAATGGA-3′ Rev 5′-CGGTCCAAGAATTTCACCTC-3′
*IFN-B*	Fwd 5′-AAACTCATGAGCAGTCTGCA-3′ Rev 5′-AGGAGATCTTCAGTTTCGGAGG-3′

### Flow cytometry

THP-1 cells were infected with VSV-G pseudotyped WT virus for 48 h. Cells were washed with phosphate-buffered saline (PBS), fixed with 4% paraformaldehyde for 10 min, permeabilized with 0.1% Tween, stained with anti-Gag KC57-FITC antibody (Beckman Coulter #6604665), and analyzed *via* flow cytometry on a BD FACS Calibur instrument.

### IFN-β ELISA assays

Cell culture supernatants were collected from infected THP-1 cells at 24 hpi and subjected to an IFN-β ELISA assay (InvivoGen, #luex-hinfbv2) following the manufacturer’s instructions. The concentration of IFN-β in cell culture supernatants was derived based on a standard curve obtained from serial dilutions of recombinant IFN-β provided by the ELISA assay.

### Fate of core assays

Analysis of retroviral cores was performed by the fate of core assay (96). Briefly, pgsA-745 cells were infected with VSV-G-pseudotyped viruses in the presence of GS-CA1 and GS-6207 (lenacapavir). After synchronized infections at 4°C, virus inoculum was removed, and cells were washed with PBS. Infected cells were incubated at 37°C for 2 h. Postnuclear supernatants were separated by ultracentrifugation on 10% to 60% linear sucrose gradients at 109,075 × *g* (30,000 rpm) for 1 h on a SW55 Ti swing-bucket rotor. Ten 500 µL fractions were collected from the top of the gradient and fractions were analyzed for vDNA by qPCR or western blotting using a mouse monoclonal antibody against capsid (NIH AIDS Reagents).

### Quantification of reverse transcription and integration in THP-1 cells

THP-1 cells were grown in six-well plates and infected with VSV-G pseudotyped WT or CA mutant viruses at an MOI of 5 in the presence of polybrene. Cells were collected at 6, 12, 18, 24, and 48 hpi, washed with PBS, and resuspended in 200 µL lysis buffer (100 mM NaCl, 10 mM Tris-HCl pH 8, 25 mM EDTA pH 8, 0.5% SDS, 0.1 mg/mL Proteinase K). Samples were incubated at 50°C by constant agitation (2,000 rpm) in a thermal mixer (Eppendorf) for 2 h. DNA was extracted by phenol-chloroform extraction. The quantity of HIV-1 RT products and 2-LTR circles was measured by qPCR using primers specific for early and late RT products (see above table). Extracted DNA was analyzed for accumulation of RT products, 2-LTR circles, and integration as described before (97, 98).

### RNAscope (FISH)

Prior to infection, 4 × 10^5^ THP-1 cells were plated in the presence of 5 µg/mL polybrene. Cells were infected with VSV-G pseudotyped viruses at an MOI of 5. At 24 hpi, cells were washed with 1× PBS and attached to Poly-L-Lysine-coated coverslips (Neuvitro #GG-12–1.5-PLL) for 10 min at 37°C. Cells were fixed with 4% PFA by incubation for 30 min at room temperature. To generate human MDMs, PBMCs from anonymous donors were stimulated with 50 ng/mL GM-CSF in RPMI 1640 Medium containing 10% fetal bovine serum for 6 days. Fresh media containing GM-CSF was provided on day 3. One day prior to infection, cells were replated in media containing 5 µg/mL polybrene in 24-well plates on Poly-L-Lysine-coated coverslips (Neuvitro #GG-12–1.5-PLL) at a density of 2 × 10^5^ cells per well. Cells were infected with VSV-G pseudotyped viruses at an MOI of 5 i.u./cell and fixed at 12 hpi with 4% PFA by incubation for 30 min at room temperature. Following fixation, cells were dehydrated, rehydrated, permeabilized, and immobilized as previously described (99). HIV-1 vDNA/RT products in cells were probed using RNAscope reagents (Advanced Cell Diagnostics #323110) per the manufacturer’s protocol with the following modifications. After the RNAscope hydrogen peroxide incubation and wash steps, samples were treated with a 1:5 dilution of Protease III for 10 min at room temperature. Samples were washed twice with PBS and subsequently treated with 25 µg/mL RNase A and 62.5 U/mL RNAse T1 (Thermo Scientific #EN0551) for 30 min at 37°C. Cells were then washed twice with PBS and incubated with probes as follows. For hybridization of the HIV-gagpol-sense target probe (Advanced Cell Diagnostics #317701), cells were covered with 50 µL probe and 1 µL formamide (Fisher Scientific), incubated at 60°C for 10 min, and then incubated in a humidified HybEZ II oven at 40°C for 21 h. Samples were washed with 0.5× wash buffer. Hybridization of AMPs 1, 2, and 3, HRP-C1 signal development, and wash steps were performed as described by the manufacturer’s protocol. RT products were labeled with a 1:1500 dilution of TSA Vivid Fluorophore 520 (Advanced Cell Diagnostics #323271). After incubating with RNAscope HRP blocker, samples were washed twice, counterstained with DAPI, washed with PBS, and mounted on slides using ProLong Gold antifade reagent. Images were taken using a Zeiss LSM 880 Airyscan confocal microscope equipped with a ×63/1.4 oil-immersion objective using the Airyscan super-resolution mode. At least 10 images were taken for each sample using the ×63 objective (THP-1s) or ×40 objective (MDMs). Numbers of nuclei and vDNA puncta in images were quantitated using Volocity 6.3 (PerkinElmer). Briefly, protocols were designed with Volocity software to separate populations of nuclei (blue) and RT products (green), and determine the intersection of these two populations (nuclear RT products).

## SUPPLEMENTAL MATERIAL

Figure S1 mbio.00348-24-s0001.pdf Supplement to Figure 1.

Figure S2 mbio.00348-24-s0002.pdf Supplement to Figure 2.

Figure S3 mbio.00348-24-s0003.pdf Supplement to Figure 3.

Figure S4 mbio.00348-24-s0004.pdf Supplement to Figure 4.

Figure S5 mbio.00348-24-s0005.pdf Supplemental to Figure 5.

Figure S6 mbio.00348-24-s0006.pdf Supplement to Figure 6.

Supplemental legends mbio.00348-24-s0007.docx Legends for supplemental figures.

ASM does not own the copyrights to Supplemental Material that may be linked to, or accessed through, an article. The authors have granted ASM a non-exclusive, world-wide license to publish the Supplemental Material files. Please contact the corresponding author directly for reuse.
