# Supplementary figures and images for: HIV-1 capsid stability and reverse transcription are finely balanced to minimize sensing of reverse transcription products via the cGAS-STING pathway
Source: mBio. 2024 Mar 26;15(5):e00348-24. doi: 10.1128/mbio.00348-24 (PMC11077976; doi:10.1128/mbio.00348-24)

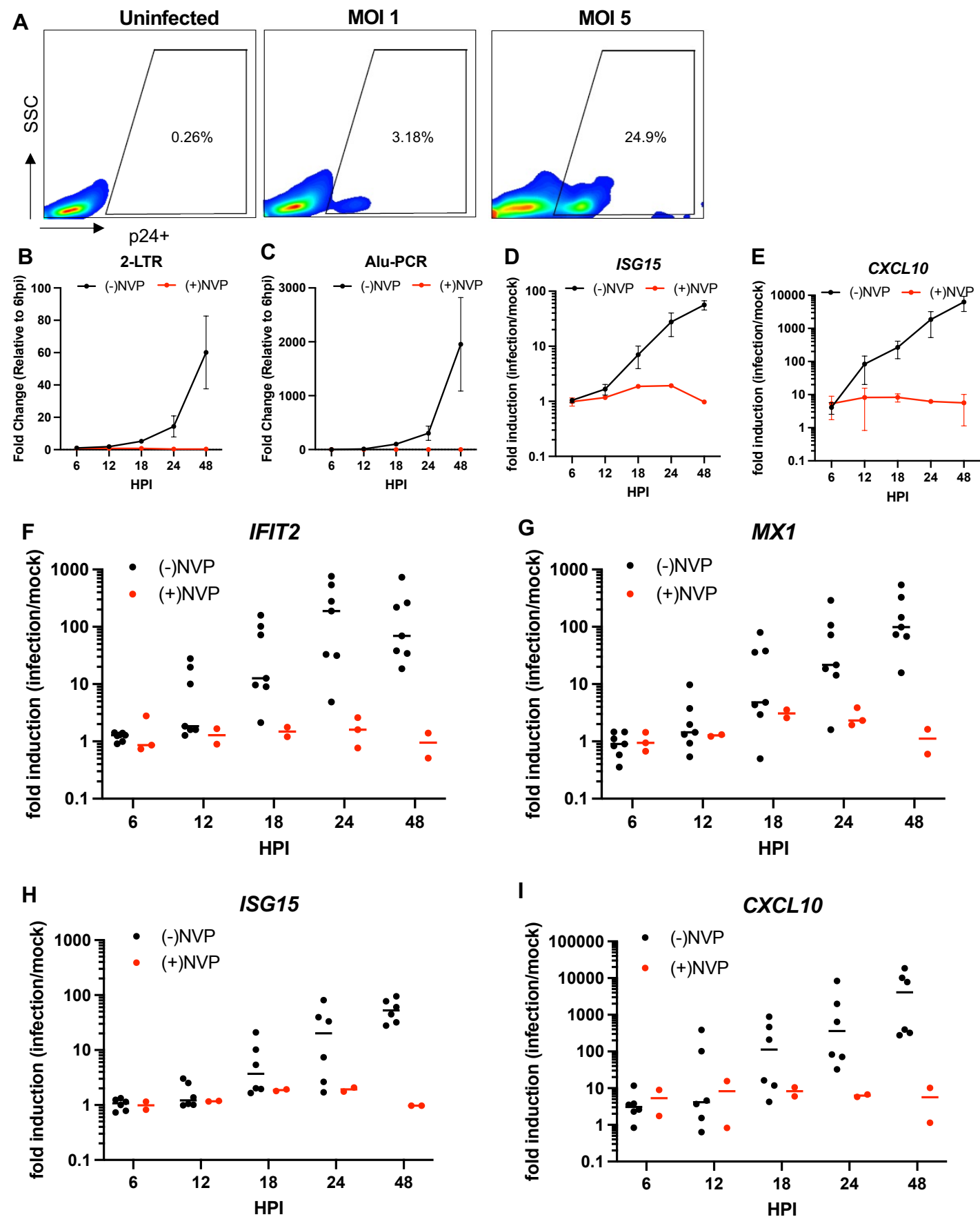

Figure S1

Supplement: Figure S1 — Supplement to Figure 1. [file mbio.00348-24-s0001.pdf]

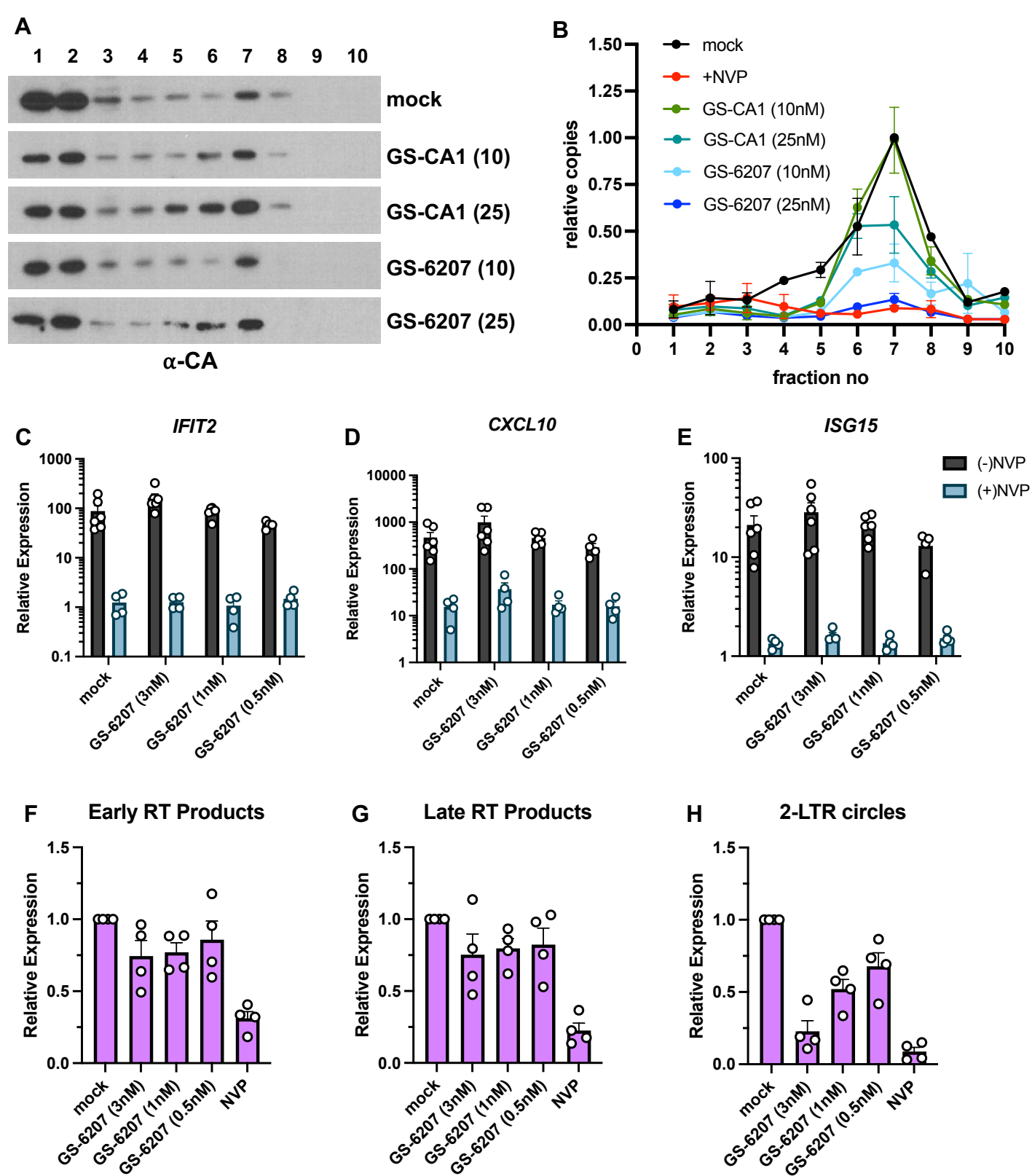

Figure S2

Supplement: Figure S2 — Supplement to Figure 2. [file mbio.00348-24-s0002.pdf]

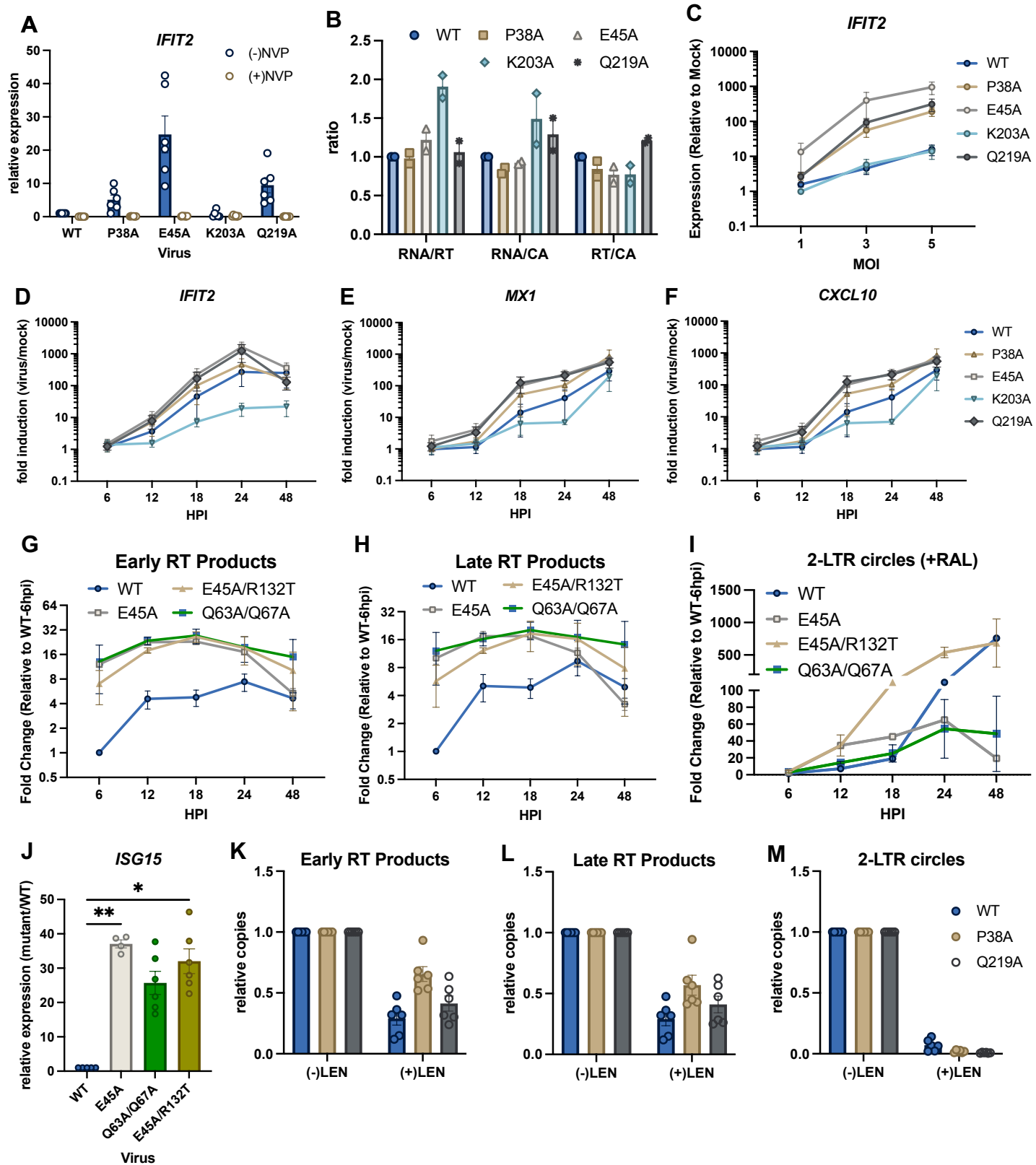

Supplement: Figure S3 — Supplement to Figure 3. [file mbio.00348-24-s0003.pdf]

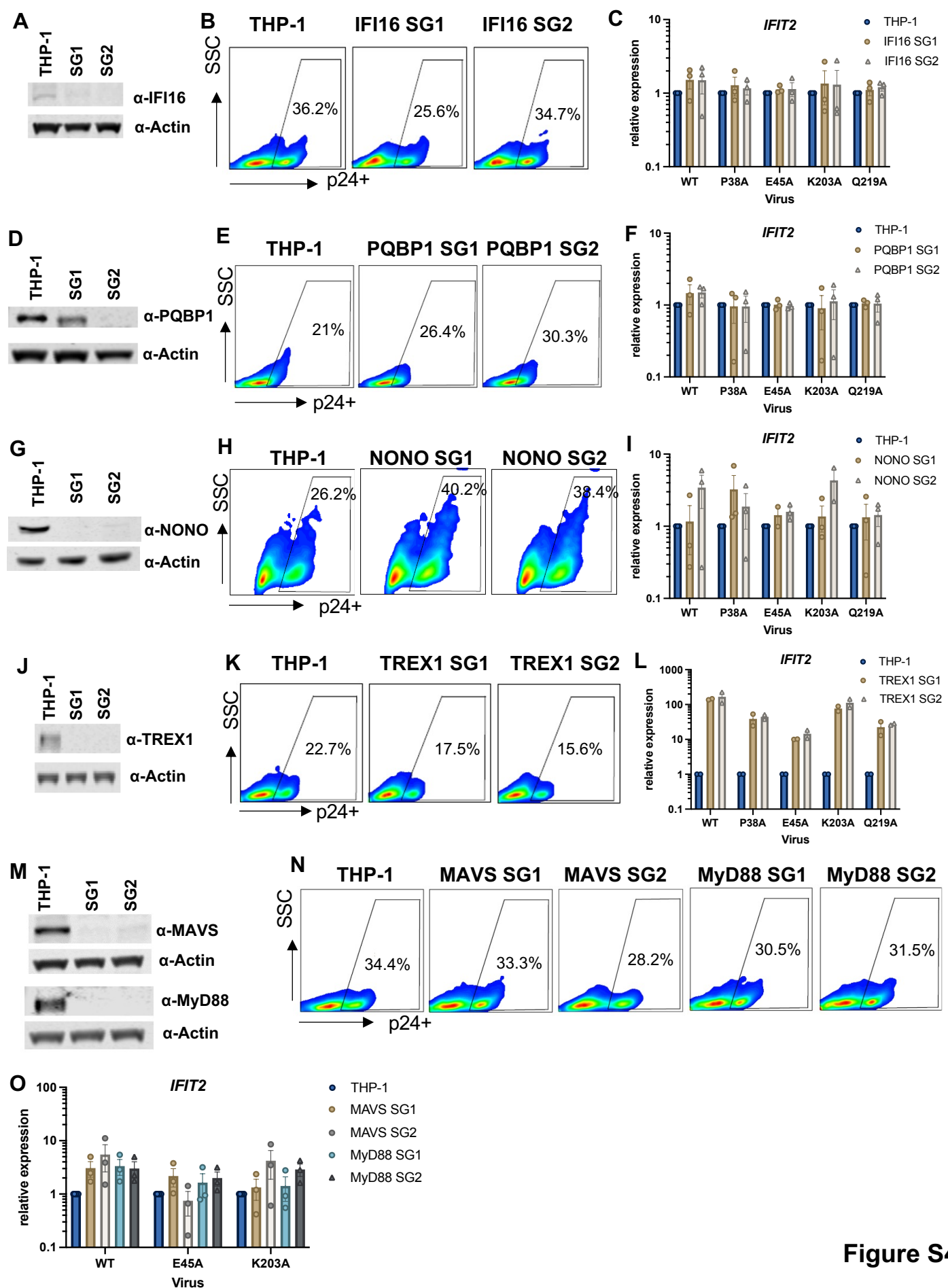

**Figure S4**

Supplement: Figure S4 — Supplement to Figure 4. [file mbio.00348-24-s0004.pdf]

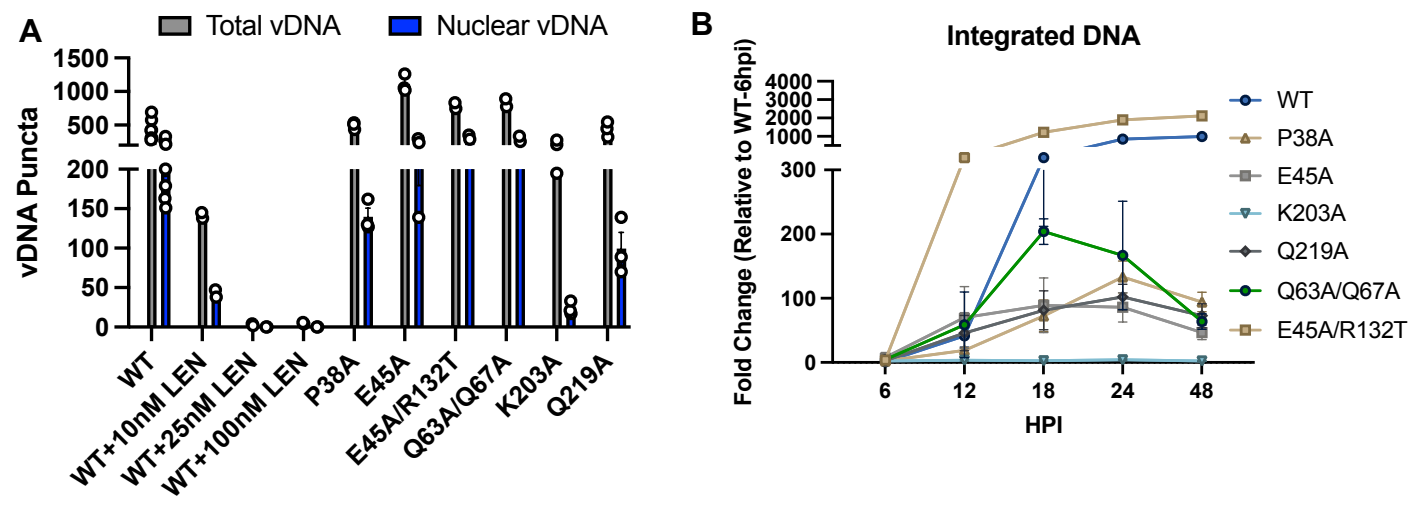

Figure S5

Supplement: Figure S5 — Supplemental to Figure 5. [file mbio.00348-24-s0005.pdf]

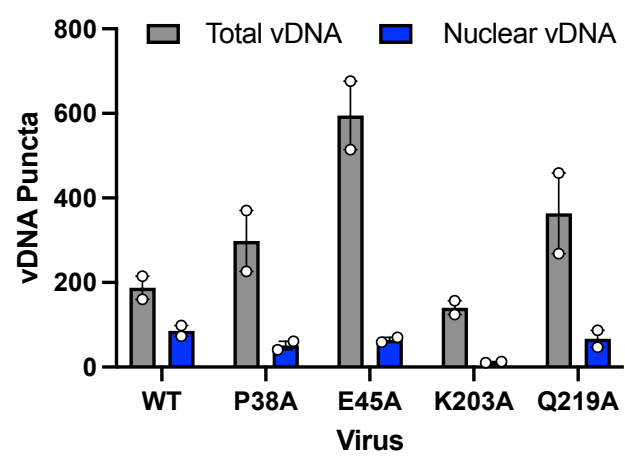

**Figure S6**

Supplement: Figure S6 — Supplement to Figure 6. [file mbio.00348-24-s0006.pdf]
