# Supplementary material for: HIV-1 capsid stability and reverse transcription are finely balanced to minimize sensing of reverse transcription products via the cGAS-STING pathway
Source: mBio. 2024 Mar 26;15(5):e00348-24. doi: 10.1128/mbio.00348-24 (PMC11077976; doi:10.1128/mbio.00348-24)
Supplement: Supplemental legends — Legends for supplemental figures. [file mbio.00348-24-s0007.docx]

**SUPPLEMENTAL FIGURE LEGENDS**

**Figure S1:** **HIV-1 reverse transcription products activate the innate immune system. (A)** THP-1 cells were infected with HIV-1_NL4-3_/VSV-G at MOI: 1 or 5 i.u./cell (based on virus titers on TZM-bL cells). Infected cells were fixed at 48 hpi, stained for p24 (CA) and analyzed by flow cytometry as detailed in Materials and Methods. (**B-I**) THP-1 cells were infected with HIV-1_NL4-3_/VSV-G at MOI: 5 i.u./cell in the absence or presence of 25μM nevirapine (NVP) as in Fig. 1. Accumulation of 2-LTR products (B), integration as assessed by Alu-PCR (C), and ISGs (D-I) were analyzed by qPCR at the indicated time points post-infection. Data in B, C were normalized relative to 6 hpi/(-)NVP samples. IFN-β and ISG expression in D-I was normalized relative to mock-infected cells. Data in panels F and G show the individual data points used in Fig. 1E, F and data in H and I show the individual data points used in panels D and E. Data are derived from n=2-7 independent biological replicates. Graphs in B-E show the mean and error bars display the SEM.

**Figure S2: Effect of lenacapavir (GS-6207) and GS-CA1 on innate immune sensing of HIV-1. (A, B)** PgsA-745 cells were synchronously infected with VSV-G-pseudotyped GFP reporter HIV-1 in the presence of 10 nM or 25nM GS-CA1 or lenacapavir (GS-6207). Cells were processed at 2 hpi, as explained in the Materials and Methods. Collected fractions were analyzed by western blotting using antibodies against CA-p24 (A) or subjected to qPCR for detection of viral reverse transcription products (B). Immunoblots in A are representative of three independent experiments and data in B show the mean (error bars denote SEM) of three independent biological replicates. (**C-H**) THP-1 cells were infected with HIV-1_NL4-3_/VSV-G at MOI: 5 i.u./cell as in Fig. 2 in the presence of 3, 1 or 0.5 nM lenacapavir (GS-6207) or 25 μM nevirapine. *IFIT2 (C)*, *CXCL10 (D)* and *ISG15 (E)* expression was analyzed at 24 hpi by qRT-PCR. A parallel set of samples were subjected to DNA extraction and accumulation of early RT products (F), late RT products (G) and 2-LTR circles (H) was analyzed at 24 hpi by qPCR. Data are derived from 4-6 independent biological replicates and normalized relative to uninfected and/or mock-treated cells. Graphs show the mean and error bars display the SEM.

**Figure S3: CA destabilizing and stabilizing mutations affect innate immune sensing of HIV-1. (A)** THP-1 cells were infected as in Fig. 3 but in the absence or presence of 25μM nevirapine (NVP). *IFIT2* expression was analyzed by qRT-PCR at 24 hpi and normalized relative to cells infected with WT HIV-1 (set to 1). Graphs show the mean derived from six independent biological replicates with error bars displaying the SEM. Note that (-) NVP conditions represent the same data presented in the main Figure 3A. **(B)** Comparison of WT vs. CA mutant virus stocks by RT activity, RNA copies and CA levels. Two independent virus stocks for each virus were analyzed for RT activity and genomic RNA copies by qRT-PCR or subjected to immunoblotting for CA-p24 whereby CA signal in standard dilutions of each virus stock was quantitated. RT activity, gRNA copies and CA levels in each virus stock was first normalized relative to WT stocks (set to 1). RNA/RT, RNA/CA and RT/CA ratios are displayed for each virus. **(C)** THP-1 cells were infected with HIV-1_NL4-3_/VSV-G at MOI: 5, 3, or 1 i.u./cell (titered on TZM-bL cells) or an equivalent particle number of CA mutants (normalized by RT activity). *IFIT2* expression was analyzed by qPCR at 24 hpi and normalized relative to mock-infected cells. Graph shows the mean derived from three independent biological replicates and error bars display the SEM. (**D-F**) THP-1 cells were infected as in panel A with the indicated CA mutant viruses. *IFIT2* (D), *MX1* (E) and *CXCL10* (F) expression was analyzed at the indicated time points post-infection by qRT-PCR. ISG expression is normalized relative to mock-infected cells. Graphs show the mean derived from three independent replicates with error bars denoting the SEM. (**G-J**) THP-1 cells were infected with HIV-1_NL4-3_/VSV-G at MOI: 5 i.u./cell (titered on TZM-bL cells) or an equivalent particle number of CA mutants (normalized by RT activity) for the indicated CA mutant viruses. Accumulation of early RT products (G), late RT products (H), 2-LTR circles (I) and *ISG15* (J) was analyzed at 24 hpi by qPCR. Induction of the indicated ISGs (J-L) was analyzed at 24 hpi by qRT-PCR and normalized relative to cells infected with WT HIV-1 (set to 1). Data in (G, H) are derived from 4, (I) from 2 and (J) from 4-6 independent replicates. Note that the E45A data set displayed here is the same as the data shown in Fig. 3H. Data show the mean and error bars display the SEM (*, p<0.05; **, p<0.01; ***, p<0.001, by one-way ANOVA multiple comparison test with Dunnett’s correction). (**K-M**) THP-1 cells were infected with the indicated viruses as above in the presence of 10 nM lenacapavir (LEN). Early RT products, late RT products and 2-LTR circles was analyzed by qPCR at 24 hpi and normalized relative to mock-treated cells for each virus. Data show the mean from 6 independent replicates, error bars display the SEM.

**Figure S4: Pathways involved in innate sensing of WT HIV-1 and CA mutants.** THP-1 cells were subjected to CRISPR-mediated knockout of IFI16 **(A-C)**, PQBP1 **(D-F)**, NONO **(G-I)**, TREX1 (**J-L**), MAVS and MyD88 **(M-O)** using two different guides (SG1 & SG2) per target. Bulk populations of the indicated cells were analyzed by immunoblotting for targeted genes to determine the knockout efficiency (A, D, G, J, M). Parental and knockout cells were infected with WT and CA mutant HIV-1/VSV-G as in Fig. 4. Infection rate with WT HIV-1/VSV-G was analyzed by flow cytometry (B, E, H, K, N). *IFIT2* induction was analyzed by qRT-PCR at 24 hpi and normalized relative to non-transduced THP-1 cells for each virus (set to 1) (C, F, I, L, O). Graphs show the mean derived from two-four independent biological replicates with error bars displaying the SEM.

**Figure S5: Accumulation and localization of RT products in infected cells.**  (**A**) Raw counts of total RT products and nuclear RT products from images from two-three independent biological replicates in THP-1 cells (as shown in Fig. 5). Graph shows the mean and error bars represent the SEM. **(B)** Infected THP-1 cells were analyzed for HIV-1 integration by Alu-PCR at indicated time points post-infection and normalized relative to cells infected with WT HIV-1 at 6 hpi (set to 1). Graphs show the mean derived from three independent biological replicates and error bars display the SEM.

**Fig. S6:** **Accumulation and localization of RT products in infected MDMs.**  Raw counts of total RT products and nuclear RT products from images from two independent biological replicates in human MDMs. Graph shows the mean and error bars represent the SEM.
